# Imaging evaluation of nano-hydroxyapatite/polyamide 66 strut in cervical construction after 1-level corpectomy: a retrospective study of 520 patients

**DOI:** 10.1186/s40001-020-00440-3

**Published:** 2020-09-01

**Authors:** Weiyang Zhong, Xinjie Liang, Xiaoji Luo, Zhengxue Quan, Dianming Jiang

**Affiliations:** 1 Department of Orthopedic Surgery, The First Affiliated Hospital of Chongqing Medical University, Chongqing, China; 2 Department of Pain Management, The First Affiliated Hospital of Chongqing Medical University, Chongqing, China; 3 Department of Orthopedic Surgery, The Third Affiliated Hospital of Chongqing Medical University, Chongqing, China

**Keywords:** Nano-hydroxyapatite/polyamide, Anterior cervical corpectomy and fusion, Imaging evaluation

## Abstract

**Background:**

The application of nano-hydroxyapatite/polyamide 66(n-HA/PA66) struts has become reliable in anterior cervical corpectomy and fusion (ACCF) as a source of sufficient segmental stability. This was a retrospective and long-term imaging evaluation of the n-HA/PA66 strut in 1-level ACCF.

**Methods:**

The patients between June 2006 and December 2014, who underwent 1-level ACCF using an n-HA/PA66 strut, were reviewed. The neurological status was assessed using the Japanese Orthopedic Association (JOA) score and axial pain was evaluated using a Visual Analogue Scale (VAS) score and the radiographic parameters were determined by X-ray and 3-D CT examinations when necessary for the evaluation of bone fusion using the Brantigan scale and imaging characteristics.

**Results:**

A total of 520 patients underwent one-level ACCF, with a mean follow-up (FU) duration of 72.38 ± 24.56 months. The level of surgery was C4 in 58 cases, C5 in 173 cases, C6 in 208 cases, and C7 in 81 cases. According to the Brantigan scale, on X-ray examination, the bony fusion rate was observed to be 40%, 70%, 93%, and 98% at 3 months, 6 months, 1 year and the final FU. An interesting radiographic appearance of the bone graft growth pattern was classified into three types. 95% of the patients accounted for types a and b. No significant differences were observed in age, hospitalization duration, surgical haemorrhage volume, or fusion rate among the types except in the percentage and sex of the patients among the types. Type a had better cervical lordosis, and less subsidence than types b and c (*P* < 0.05). No significant difference was found in segment angle between type a and type b. Type c was more often observed with subsidence rate, segmental angle loss and cervical alignment loss than types a and b (*P* < 0.05). Type a also had a slightly higher fusion rate, than types b and c, but there were no significant differences. The overall mean JOA score at the final follow-up among the groups were significantly improved comparing that preoperatively and no significant differences were found among the groups, no matter pre-operation or final follow-up. The overall mean VAS score at the final follow-up among the groups were significantly improved comparing that preoperatively and no significant differences in preoperative VAS score were found among the groups. However, the VAS score at the final follow-up of type a or type b was better than type c. No patients received revision surgery.

**Conclusions:**

The type a bone graft growth pattern could allow a lower incidence of subsidence and better maintenance of local and global alignment to be achieved and is thus proposed for surgeons.

## Background

As an alternative technique for spine surgeons, ACCF can allow satisfactory decompression and stable reconstruction of the cervical spine to be achieved in treating cervical trauma, cervical spondylotic myelopathy (CSM), and cervical kyphosis [1–3]. The n-HA/PA66 strut has become reliably applied for reconstruction in ACCF to achieve sufficient, fast segmental stability and assure bone graft fusion; additionally, the strut can be filled with autograft, allograft, or artificial bone graft material. However, ensuring satisfactory bone fusion and avoiding implant-related complications are key, no matter which cage is used [4, 5]. Hence, radiographic follow-up examinations are very important for evaluating cages and struts to help surgeons improve and shorten the learning curve.

However, there are still no uniform, non-invasive, standard strategies for bone graft fusion. X-ray examination is usually used for evaluation, although there are several assessment methods, and despite stress shielding, postoperative radiographic interference is still of great concern [6, 7].

In our study, we used X-ray and 3-D computed tomography (CT) examinations when necessary with the Brantigan scale [12] to observe the radiographic characteristics of bone fusion with the n-HA/PA66 strut in cervical reconstruction to devise a system for follow-up (FU) evaluation.

## Materials and methods

### Sample selection

**Fig. 1:**
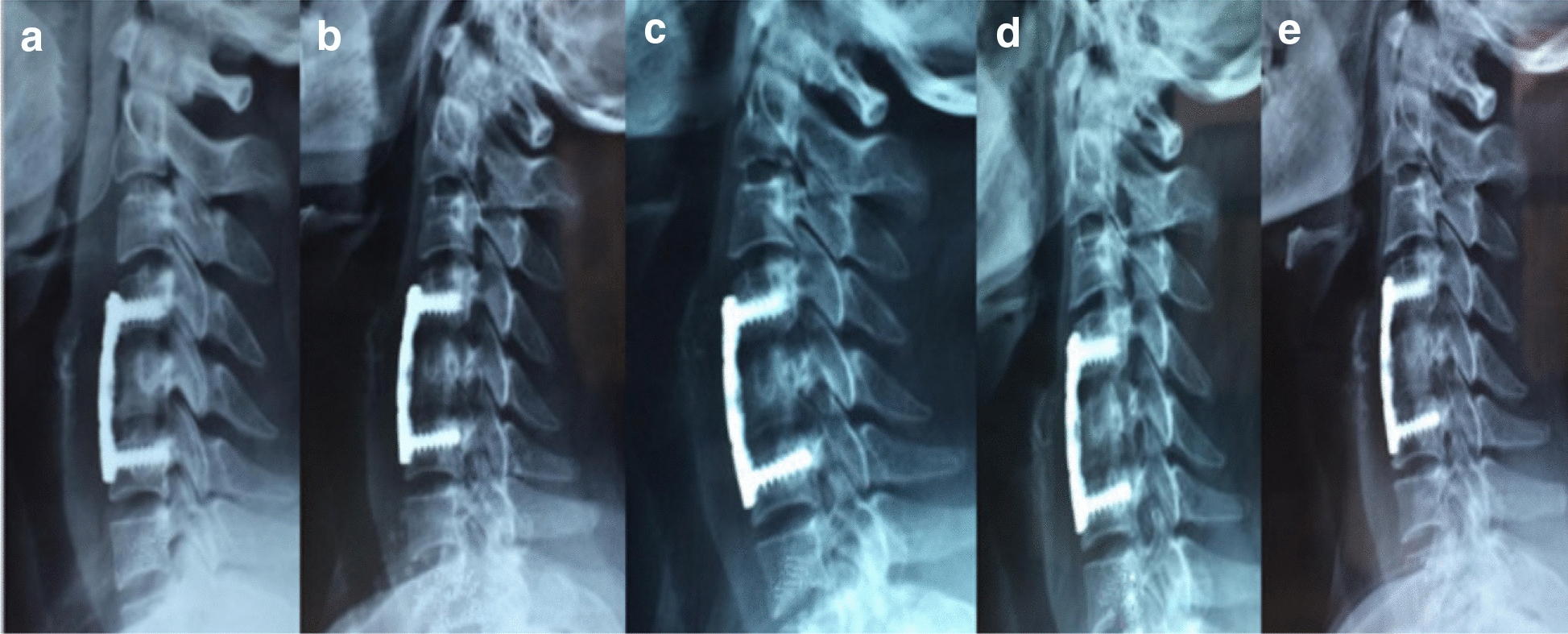
Osseous fusion according to the Brantigan scale. **a**–**e** Mean grade of 1–5 in the n-HA/PA66 strut

Between June 2006 and December 2014, 520 patients who underwent one-level ACCF with a locking titanium plate performed by the same spine team were retrospectively reviewed. All patients manifested symptoms of CSM, and radiographic data facilitated the diagnosis along with X-ray, magnetic resonance imaging (MRI), and 3-D CT examinations when necessary. The exclusion criteria were severe kyphosis, infection, and tumour. The n-HA/PA66 strut was designed and fabricated by the Institute of Materials Science and Technology, Sichuan University, and our department. Nano-hydroxyapatite/polyamide66 (n-HA/PA66) is a biomimetic composite synthesized from nano-scale HA and the polar polymer PA66 (Fig. 1) which was approved for clinical use in 2005 by the State Drug and Food Administration of China and The n-HA/PA66 are produced by Sichuan Guona Technology Co., LTD.

### Surgical technique

A right-incision anterior cervical approach was performed by the senior spine surgeon for all patients. After accurate exposure and adequate distraction using a Caspar screw, 1-level corpectomy was performed, and the dura mater was widely decompressed. A suitably sized n-HA/PA66 strut filled with the resected vertebra was implanted using a titanium anterior cervical plate (Johnson and Johnson, Co., Depuy Spine, Ltd., Raynham, MA, U.S.A.). After the operation, all patients were advised to wear a Philadelphia hard cervical collar for 6–8 weeks. Furthermore, the patients began step-by-step neck muscle training.

### Outcome assessment

**Fig. 2:**
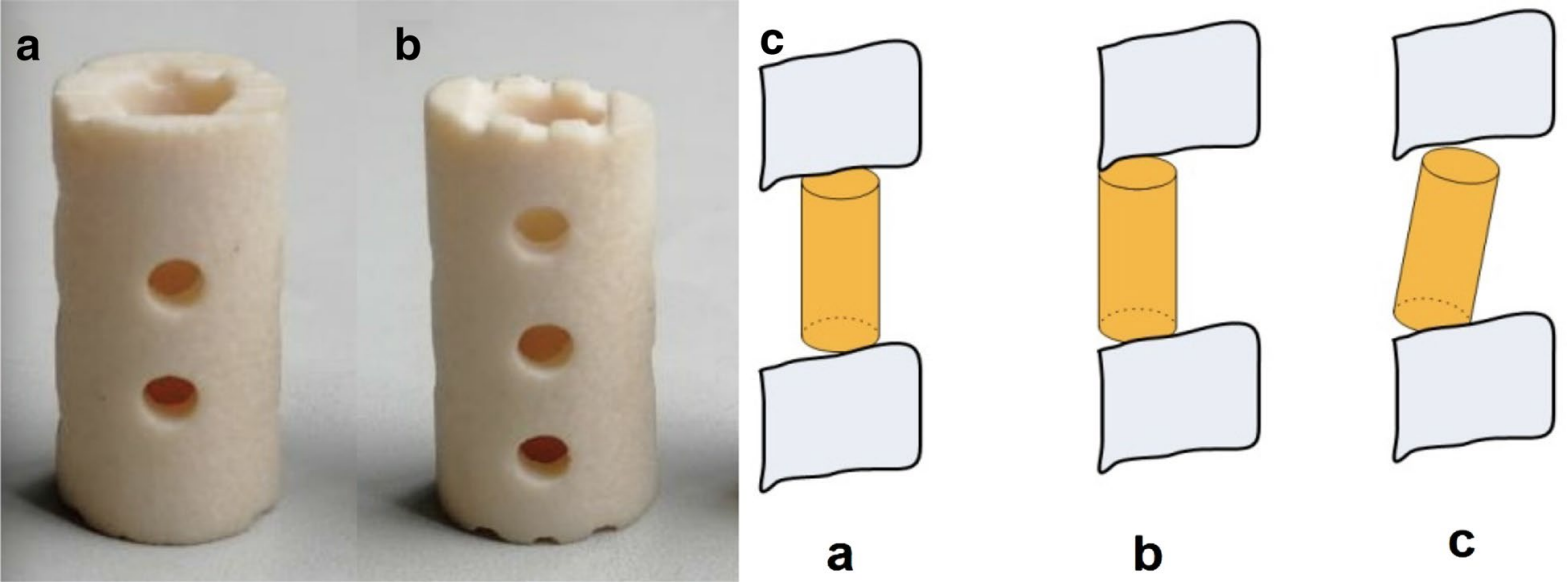
Frontal (**a**) and lateral and view photographs (**b**) of a n-HA/PA66 strut and the depiction of the three bone graft growth patterns for the n-HA/PA66 strut (**c**)

Radiographic FU examinations, including X-ray and 3-D CT (when necessary, usually only at final FU), were performed immediately and at 3 months, 6 months and 12 months postoperatively and then annually thereafter. The bone graft fusion status was assessed based on the X-ray film or 3-D CT scan and ranked according to the 5 grades defined by Brantigan et al. [12] (Fig. 1). The Japanese Orthopedic Association (JOA) score was used to evaluate neurologic status, and the visual analogue scale (VAS) was used to assess arm and neck pain. The radiographic evaluation of bony fusion was performed by two independent observers (a senior spine surgeon and an attending radiologist who had not participated in the surgeries). We defined grades greater than 3 on the Brantigan scale as bone fusion, and the bone graft growth pattern was recorded at the final follow-up visit and the bone graft growth pattern was defined as the direction of bone growth and the strut. On the X-ray films, the bone graft growth pattern of the n-HA/PA66 strut was defined as one of three types: a: the growth direction is along the middle column to the upper and lower vertebral bodies; b: the growth direction is along the anterior column; and c: the growth direction is along the middle column of the upper vertebral body to the anterior column of the lower vertebral body (Fig. 2). The anterior column was defined as anterior 1/2 of vertebral body and the middle column as posterior 1/2 of vertebral body. Subsidence was defined as a decrease of more than 3 mm in the fused intervertebral height during the follow-up period. According to the Cobb method, cervical lordosis was defined as the angle formed between the inferior endplates of C2 and C7, and the angle of the segmental fused segments was defined as the angle formed between the superior endplate of the upper vertebral body and the inferior endplate of the lower vertebral body.

### Statistical analysis

All statistical data were analysed using Statistic Analysis System software (SAS Institute, Inc., Cary, NC, USA). Quantitative variables are described as the mean ± SD. Categorical variables were analysed by Chi-squared test. Values of *P* < 0.05 were regarded as statistically significant.

## Results

A total of 520 patients underwent one-level ACCF, with a mean FU duration of 72.38 ± 24.56 months. The level of surgery was C4 in 58 cases, C5 in 173 cases, C6 in 208 cases, and C7 in 81 cases. The distribution of Brantigan grades determined radiographically different time points is shown in Table 1. According to the Brantigan scale, on the X-ray images, the bony fusion rate was 40%, 70%, 93%, and 98% at the 3-month, 6-month, 1-year and final follow-up visit, respectively.

**Table 1:** Brantigan grade distribution determined radiographically at different time points

	3 months post-op	6 months post-op	12 months post-op	Final FU
Grade 1				
Grade 2	60%	10%		
Grade 3	40%	20%	3%	1%
Grade 4		50%	80%	10%
Grade 5			10%	87%

**Fig. 3:**
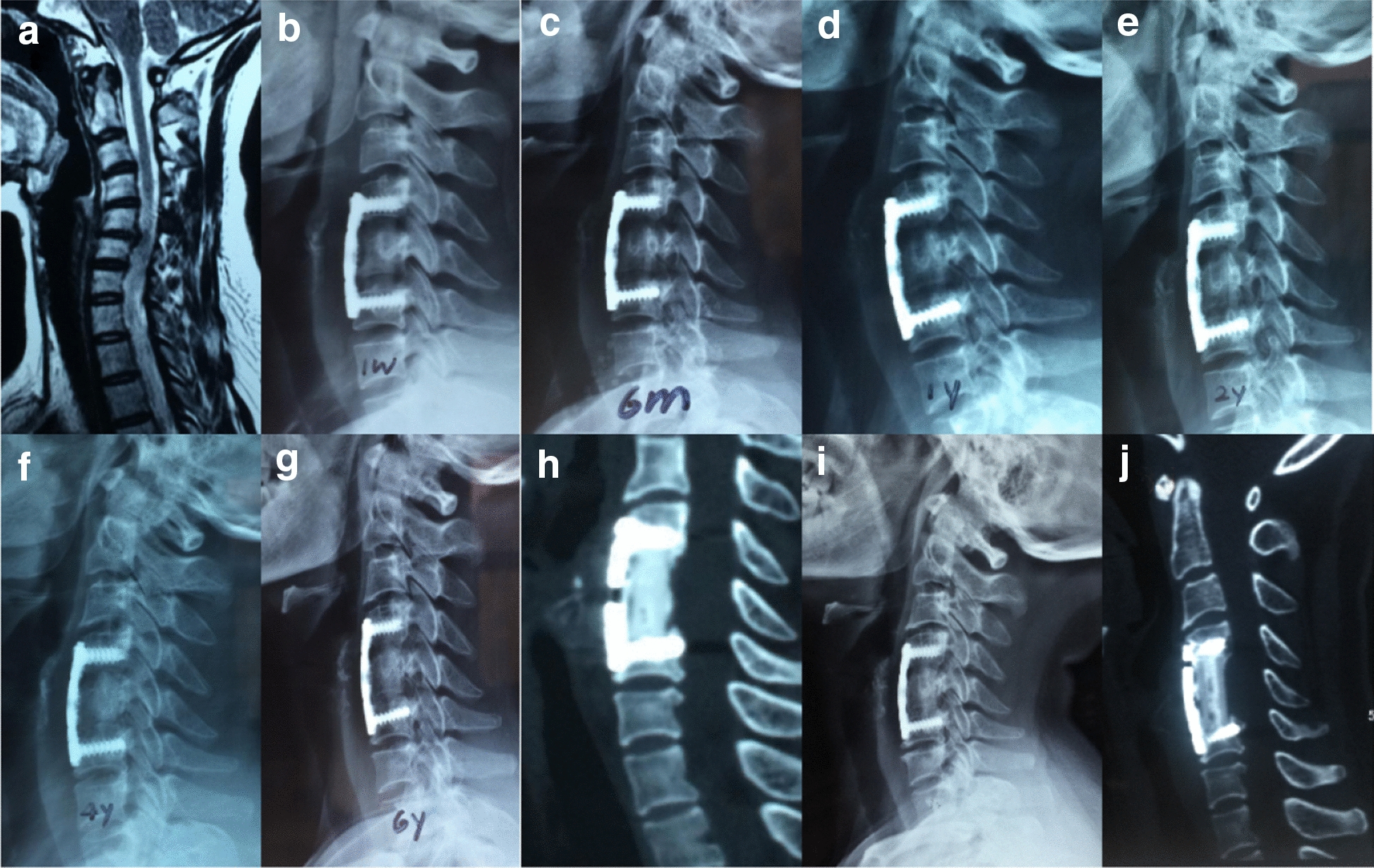
Type a bone fusion. **a**–**k** A 43-year-old woman whose preoperative cervical MRI (**a**) revealed C5/6 disc herniation underwent 1-level corpectomy with an n-HA/PA66 strut (**b**). Lateral X-ray films indicated bony fusion of the autogenous bone granules filling the strut at the 3-month, 6-month, 12-month, 2-year, 4-year, 6-year, and 8-year follow-up visits (**c**–**g**, **i**). 3-D CT at the 6-year and 8-year follow-up visits (**h**, **j**) showed solid bone fusion

**Fig. 4:**
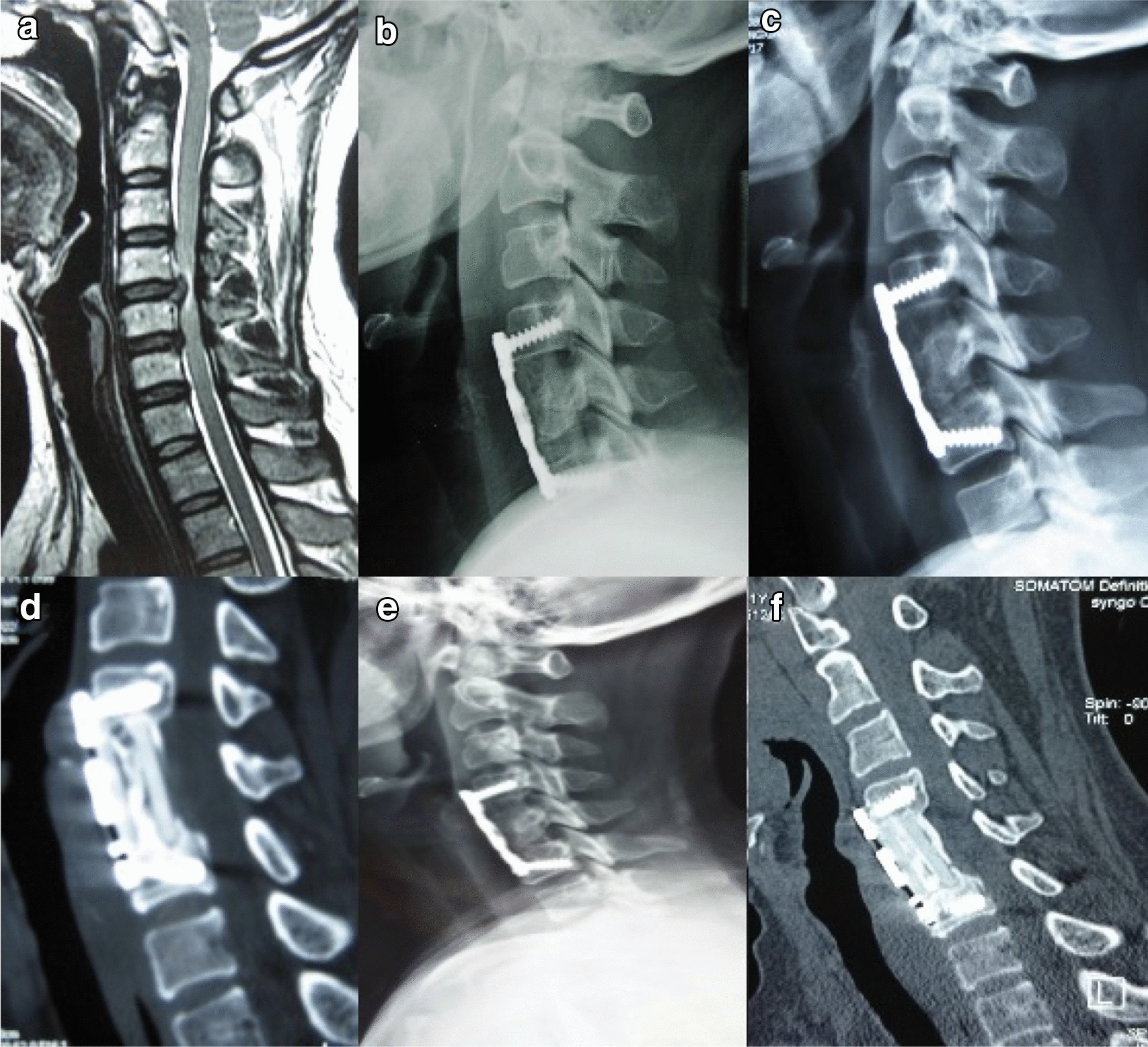
Type b bone fusion. **a**–**f** A 41-year-old woman whose preoperative cervical MRI (**a**) revealed C4/5 disc herniation compressing the spinal cord underwent 1-level corpectomy with an n-HA/PA66 strut. Lateral X-ray films indicated bony fusion of the autogenous bone granules filling the strut, and subsidence was observed at the 6-month, 12-month, and 8-year follow-up visits (**b**, **c**, **e**). 3-D CT at the 3-year and 8-year follow-up visits (**d**, **f**) showed solid bone fusion

**Fig. 5:**
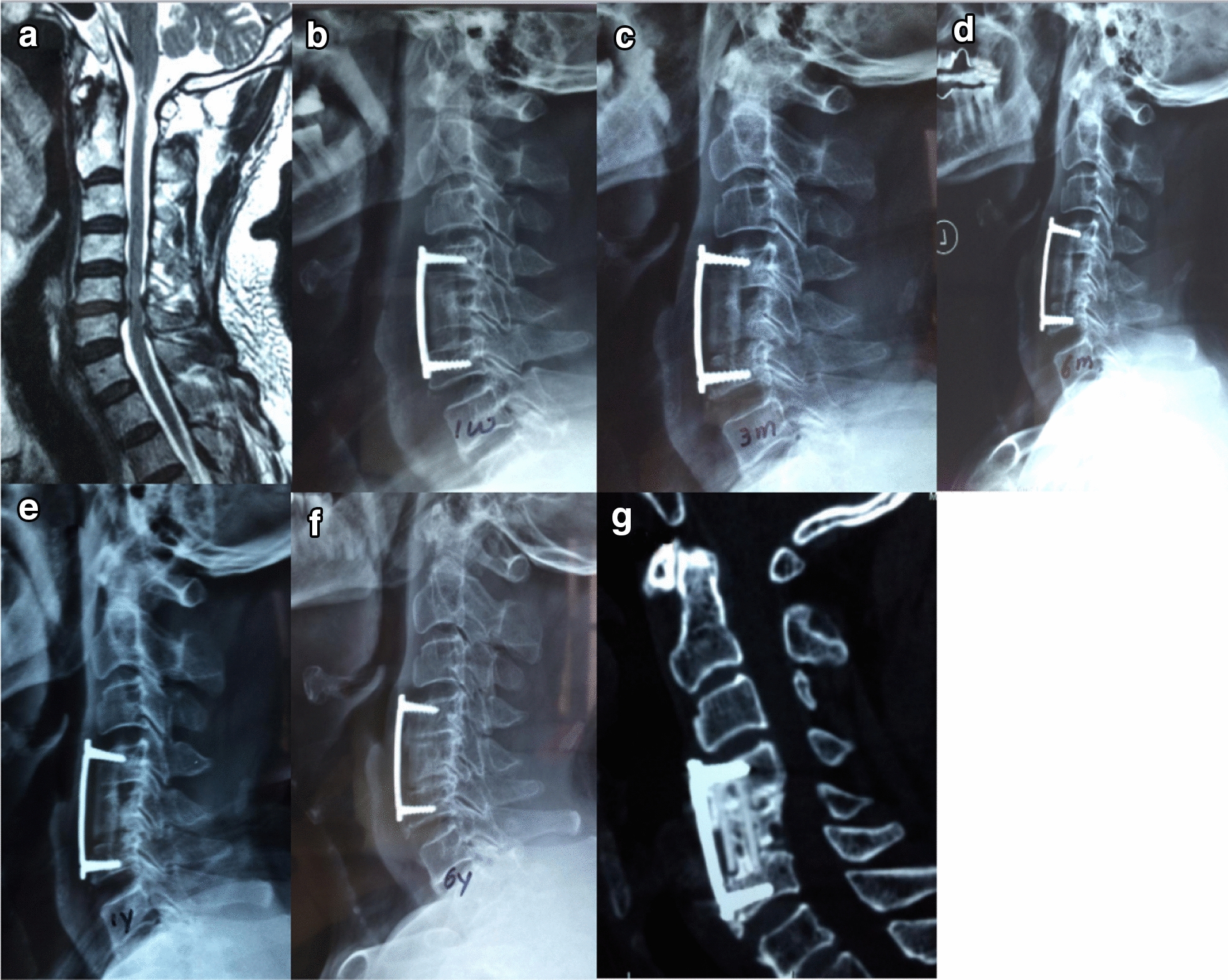
Type c bone fusion. **a**–**f** A 73-year-old man whose preoperative cervical MRI (**a**) revealed C5/6 disc herniation underwent 1-level corpectomy with an n-HA/PA66 strut (**b**). Lateral X-ray films indicated bony fusion of the autogenous bone granules filling the strut. Obvious subsidence and screw displacement were observed at the 3-month, 6-month, 12-month, and 6-year follow-up visits (**c**–**f**). 3-D CT at the 6-year follow-up (**g**) showed solid bone fusion, and the patient complained of no symptoms or signs

Furthermore, we found an interesting appearance of the bone graft growth pattern on imaging, which was classified into three types (Fig. 2). There were no statistically significant differences in age, hospitalization duration, operative duration, surgical haemorrhage, drainage volume, or fusion rate among the groups except in the percentage and sex of the patients. All patients were analysed according to the graft pattern (Table 2). In all, 95% of the patients accounted for types a and b. Type a had a slightly higher fusion rate than type b and c (*P* > 0.05) and better cervical alignment, less subsidence rate than type b and c (*P* < 0.05). However, no significant difference was found in segment angle between type a and type b (*P* > 0.05). Type c was more often observed more with subsidence rate, segmental angle loss and cervical alignment loss than types a and b (*P* < 0.05). Moreover, if a patient with the type a or b pattern showed a grade of 3 or 4 on the Brantigan scale or even non-union, progression to type c could occur (Figs. 3, 4, 5).

**Table 2:** Radiographic data of bone graft growth pattern

	a	b	c	*P* ab		*P* ac	*P* bc
Percent of patients	20% (104/520)	75% (390/520)	5% (26/520)	< 0.001	< 0.001		< 0.001
Males/females (*n*)	45/59	211/179	15/11	< 0.001	< 0.001		< 0.001
Mean age	51.85 ± 10.30	56.67 ± 9.06	58.40 ± 8.22	0.7467	0.3389		0.1248
Hospitalization duration (days)	9.25 ± 2.05	8.76 ± 1.99	9.00 ± 2.70	0.1705	0.6479		0.1019
Operative duration (min)	95.25 ± 17.66	99.73 ± 13.10	91.5 ± 15.47	0.5716	0.7616		0.2944
Surgical haemorrhage (ml)	82.75 ± 12.43	93.40 ± 13.86	90.0 ± 20.28	0.0857	0.3105		0.7017
Drainage (ml)	17.00 ± 6.77	17.58 ± 6.17	18.00 ± 7.15	0.9599	0.9515		0.9236
Fusion rate	99%	98%	97%	0.9687	0.9268		0.9715
Subsidence rate	2%	5%	10%	0.0015	< 0.001		< 0.001
Segmental angle (°)	6.16 ± 3.67	7.06 ± 5.67	12.36 ± 6.78	0.4914	< 0.001		< 0.001
C2–C7 Cobb angle (°)	1.57 ± 20.55	5.66 ± 18.05	7.87 ± 13.55	0.023	< 0.001		< 0.001

The overall mean JOA score at the final FU among the groups were significantly improved comparing that preoperatively (*P* < 0.0001) and no significant differences were found among the groups no matter pre-operation or final follow-up. The overall mean VAS score at the final FU among the groups were significantly improved comparing that preoperatively (*P* < 0.0001) and no significant differences in preoperative VAS score were found among the groups. However, the VAS score at the final follow-up of type a or type b was better than type c (*P* < 0.05) (Table 3). According the VAS score at the final FU of type c, the imaging may explain clinical outcomes.

**Table 3:** Clinical data of bone graft growth pattern

	a	b	c	*P* ab	*P* ac	*P* bc
JOA score						
Pre-operation	11.22 ± 1.39	11.67 ± 1.32	11.53 ± 1.89	0.4978	0.8512	0.9153
Final FU	16.00 ± 0.86	15.78 ± 0.67	16.11 ± 0.78	0.5504	0.6549	0.7733
VAS score						
Pre-operation	6.00 ± 0.82	5.70 ± 0.82	5.90 ± 0.88	0.4240	0.7202	0.8635
Final FU	1.40 ± 0.52	1.30 ± 1.50	2.20 ± 0.79	0.6601	0.0049	0.0065

3-D CT images evaluated using the Brantigan scale showed 99% bone fusion. Regarding the results obtained by 3-D CT and X-ray examinations, statistical analysis demonstrated a kappa value of 0.90, a *χ*^2^ value of 0.35, and *P* > 0.05. The data showed no significant difference in the bone fusion rate evaluated by X-ray and 3-D CT reconstruction, and the data were highly consistent.For the two observers, the rates of the correct diagnosis, sensitivity and specificity were 96.2%, 85.9% and 95.5%, respectively.

## Complications

Some postoperative complications occurred, such as incision superficial hematoma (one case in group of type a, two cases in group of type b and one case in group of type c) which were healed by drainage and dressing changes, superficial infection (three cases in group of type b) which were healed by dressing changes and oral antibiotics, recurrent laryngeal nerve injury (one case in group of type a) which recovered to normal at 3 months FU.

## Discussion

In recent years, anterior decompression and bone graft fusion have become popular as reliable and effective procedures for the management of cervical degeneration, tumours, trauma and other diseases [8–11]. The advantage of direct decompression of the spinal cord has been confirmed by the successful clinical outcomes reported in many studies. Interbody fusion cages can provide good postoperative stability. The use of cervical interbody fusion cages combined with internal plate fixation can improve the rate of bone graft fusion, which is gradually becoming widely used in clinical practice [12].

There is always great concern regarding the definition of bone graft fusion. X-ray films were used to evaluate bony fusion postoperatively, and bone graft growth could be directly observed. In our study, the Brantigan scale, with five grades, showed strict assessment when applied to the X-ray films, which was highly consistent with the results derived from CT reconstruction. Although 3-D CT reconstruction is relatively expensive, upon the suspicion of implant loosening or non-union, it could improve the rate of early diagnostic accuracy. Hence, X-ray was performed in all patients and 3-D CT examinations while necessary to detect the fusion status. The rate of bone fusion was 40%, 70%, 93%, and 99% at the 3-month, 6-month, 1-year and final follow-up visits. Slight pseudarthrosis could easily be observed. Precise fusion of the n-HA/PA66 strut based on 3-D CT and on radiography was accurately followed [14–19].

In our research, three types of bone graft growth patterns were observed on the X-ray films and CT images. In all, 95% of the patients accounted for types a and b, which had a lower subsidence rate and more anatomical global and local cervical alignment than type c. Furthermore, at the final FU, the VAS score of type a or type b was better than type c. The imaging may explain clinical outcomes. Although type c had poorer radiographic outcomes, the bony fusion rate was similar for types a and c, which is why the patients did not manifest symptoms or only exhibited slight neck pain that could be treated with drugs or physiotherapy [12, 13]. The reason for the most cases being classified as type b is probably the relatively narrowing of the middle column caused by the intraoperative over-extension position and the surgeons fearing that implant displacement could lead to compression of the spinal cord. Therefore, smaller diameter struts were preferred and were safer and more efficacious. Moreover, if a patient with a type a or b pattern showed a grade of 3 or 4 on the Brantigan scale or even non-union, progression to type c is likely and requires close follow-up observation. It means that a possible progression from type a to type c indicates an insufficient bony fusion. Once a type c pattern is observed, strict bracing of the cervical spine or even revision surgery will need to be considered [16–19]. However, no signs of plate or screw loosening were observed and no patients received revision surgery.

The present study, for the first time, investigated the imaging characteristics of an n-HA/PA66 strut in one-level ACCF. However, our study has a few limitations. First, the retrospective nature of the study indicates the possibly of bias. Second, because CT examinations were not routine for each follow-up visit, there may have been deviation from the bone fusion observations derived from the X-ray films, although there were two independent observers. Third, the bone growth patterns depend on surgical techniques of the surgeons which were with bias. Fourth, bone graft fusion was assessed using only the Brantigan scale and although the Brantigan scale is not commonly used in cervical surgery, its relatively carefully graded through literatures review and comparison with other criterias. Therefore, we adopt this assessment. But here may be a selective bias, and more prospective studies are needed to confirm the reliability and more criterias for bone fusion should be considered in the future. Fifth, the growth patterns covered only the sagittal plane, although the strut was implanted in the center between the vertebral body under direct vision and there no significant differences of strut growth patterns among the groups, more patients need to be followed up.

## Conclusion

The long-term results of imaging the n-HA/PA66 strut showed that 95% of the patients accounted for the type a and b bone graft growth patterns; additionally, the type a pattern could allow a lower incidence of subsidence, better maintenance of local and global alignment to be achieved and is thus proposed for surgeons.

## Data Availability

The datasets used and/or analyzed during the current study are available from the corresponding author on reasonable request.

## Abbreviations

n-HA/PA66: Nano-hydroxyapatite/polyamide 66
ACCF: Anterior cervical corpectomy and fusion
CSM: Cervical spondylotic myelopathy
CT: Computed tomography
MRI: Magnetic resonance imaging
FU: Follow-up

## References

[1] Smith GW, Robinson RA (1958). The treatment of certain cervical-spine disorders by anterior removal of the intervertebral disc and interbody fusion. Bone Joint Surg. Am.

[2] Bohlmann HH (1981). Surgical techniques of anterior decompression and fusion for spinal cord injuries. Clin Orthop.

[3] Cloward RB (1988). The anterior surgical approach to the cervical spine: the Cloward Procedure: past, present, and future. Spine.

[4] Bayerl SH, Pöhlmann F, Finger T (2018). Two-level cervical corpectomy-long-term follow-up reveals the high rate of material failure in patients, who received an anterior approach only. Neurosurg Rev.

[5] Niedzielak TR, Palmer J, Malloy JP (2018). Clinical comparison of surgical constructs for anterior cervical corpectomy and fusion in patients with cervical spondylotic myelopathy or ossified posterior longitudinal ligament: a systematic review and meta-analysis. Clin Spine Surg.

[6] Zeng J, Duan Y, Yang Y (2018). Anterior corpectomy and reconstruction using dynamic cervical plate and titanium mesh cage for cervical spondylotic myelopathy: a minimum 5-year follow-up study. Medicine.

[7] Qin R, Chen X, Zhou P (2018). Anterior cervical corpectomy and fusion versus posterior laminoplasty for the treatment of oppressive myelopathy owing to cervical ossification of posterior longitudinal ligament: a meta-analysis. Eur Spine.

[8] Yamauchi K, Fushimi K, Miyamoto K (2017). Sagittal alignment of a strut graft affects graft subsidence and clinical outcomes of anterior cervical corpectomy and fusion. Asian Spine J.

[9] Lu T, Liu C, Yang B (2017). Single-level anterior cervical corpectomy and fusion using a new 3D-printed anatomy-adaptive titanium mesh cage for treatment of cervical spondylotic myelopathy and ossification of the posterior longitudinal ligament: a retrospective case series study. Med Sci Monit.

[10] Lu T, Liang H, Liu C (2017). Effects of titanium mesh cage end structures on the compressive load at the endplate interface: a cadaveric biomechanical study. Med Sci Monit.

[11] Weber MH, Fortin M, Shen J (2017). Graft subsidence and revision rates following anterior cervical corpectomy: a clinical study comparing different interbody cages. Clin Spine Surg.

[12] Brantigan JW, Steffee AD (1993). A carbon fiber implant to aid interbody lumbar fusion. Two year clinical results in the first 26 patients. Spine (Phila Pa 19766).

[13] Eck KR, Bridwell KH, Ungacta FF (2000). Analysis of titanium mesh cages in adults with minimum two-year follow-up. Spine (Phila Pa 19766).

[14] Zeng Z, Dian J, Yun O (2012). A hollow cylindrical nano-hydroxyapatite/polyamide composite strut for cervical reconstruction after cervical corpectomy. Clin Neurosci.

[15] Yang X, Chen Q, Liu L (2013). Comparison of anterior cervical fusion by titanium mesh cage versus nano-hydroxyapatite/polyamide cage following single-level corpectomy. Int Orthop.

[16] Zhang Y, Deng X, Jiang D (2016). Long-term results of anterior cervical corpectomy and fusion with nano-hydroxyapatite/polyamide 66 strut for cervical spondylotic myelopathy. Sci Rep.

[17] Lau D, Song YH, Guan Z (2013). Radiological outcomes of static vs. expandable titanium cages after corpectomy: a retrospective cohort analysis of subsidence. Neurosurgery.

[18] Zhang Y, Quan Z, Zhao Z (2014). (2014) Evaluation of anterior cervical reconstruction with titanium mesh cages versus nano-hydroxyapatite/polyamide66 cages after 1- or 2-level corpectomy for multilevel cervical spondylotic myelopathy: a retrospective study of 117 patients. PLoS ONE.

[19] Zhong W, Liang X, Tang K (2018). Nanohydroxyapatite/polyamide 66 strut subsidence after one-level corpectomy: underlying mechanism and effect on cervical neurological function. Sci Rep.

